# Evaluation of immunological escape mechanisms in a mouse model of colorectal liver metastases

**DOI:** 10.1186/1471-2407-10-82

**Published:** 2010-03-07

**Authors:** Martin Grimm, Martin Gasser, Marco Bueter, Johanna Strehl, Johann Wang, Ekaterina Nichiporuk, Detlef Meyer, Christoph T Germer, Ana M Waaga-Gasser, Andreas Thalheimer

**Affiliations:** 1 Department of General Surgery, University of Wuerzburg Hospital, Germany; 2 Department of General Surgery, Molecular Oncology and Immunology, University of Wuerzburg Hospital, Germany; 3 Department of General Surgery, Leopoldina Hospital, Schweinfurt, Germany

## Abstract

**Background:**

The local and systemic activation and regulation of the immune system by malignant cells during carcinogenesis is highly complex with involvement of the innate and acquired immune system. Despite the fact that malignant cells do have antigenic properties their immunogenic effects are minor suggesting tumor induced mechanisms to circumvent cancer immunosurveillance. The aim of this study is the analysis of tumor immune escape mechanisms in a colorectal liver metastases mouse model at different points in time during tumor growth.

**Methods:**

CT26.WT murine colon carcinoma cells were injected intraportally in Balb/c mice after median laparotomy using a standardized injection technique. Metastatic tumor growth in the liver was examined by standard histological procedures at defined points in time during metastatic growth. Liver tissue with metastases was additionally analyzed for cytokines, T cell markers and Fas/Fas-L expression using immunohistochemistry, immunofluorescence and RT-PCR. Comparisons were performed by analysis of variance or paired and unpaired *t *test when appropriate.

**Results:**

Intraportal injection of colon carcinoma cells resulted in a gradual and time dependent metastatic growth. T cells of regulatory phenotype (CD4+CD25+Foxp3+) which might play a role in protumoral immune response were found to infiltrate peritumoral tissue increasingly during carcinogenesis. Expression of cytokines IL-10, TGF-β and TNF-α were increased during tumor growth whereas IFN-γ showed a decrease of the expression from day 10 on following an initial increase. Moreover, liver metastases of murine colon carcinoma show an up-regulation of FAS-L on tumor cell surface with a decreased expression of FAS from day 10 on. CD8+ T cells express FAS and show an increased rate of apoptosis at perimetastatic location.

**Conclusions:**

This study describes cellular and macromolecular changes contributing to immunological escape mechanisms during metastatic growth in a colorectal liver metastases mouse model simulating the situation in human cancer.

## Background

Colorectal carcinoma is the third most common cause of cancer-related deaths worldwide. Although great proceedings have been made in diagnosis and treatment, still 40-50% of colorectal cancer patients die of the disease within five years of diagnosis [1]. Formation of hepatic metastases in colorectal cancer is associated with poor prognosis, resulting in the death of more than 80% of patients over the long-term [2]. Surgery is the primary treatment option for isolated metastases, but only 20% to 25% of patients displaying hepatic metastases are suitable for resection and recurrence after surgical therapy is frequent [3]. Therefore, the development of new treatment modalities for liver metastases of colorectal cancer is urgently needed. Particularly with regard to therapeutic modification of the immune system, understanding of tumor-specific immunological responses and mechanisms leading to induction or suppression of the immune system are required.

The local and systemic activation and regulation of the immune system by malignant cells during carcinogenesis is highly complex with involvement of the innate and acquired immune system [4]. Despite the fact that malignant cells do have antigenic properties their immunogenic effects are minor. The causes of a weak immune response to malignant cells are multifarious and subsumed in the term "tumor immune escape". Important single mechanisms of the immune escape are down regulation of MHC-class I complex, loss of co-stimulatory surface antigens, decreased expression of apoptosis inducing death receptors (e.g. Fas/TRAIL receptor) on malignant cell, and loss of tumor infiltrating cytotoxic T cells by tumor induced apoptosis [5]. An additional, very important aspect of the "tumor immune escape" during carcinogenesis is a significant disturbed cellular immune response [6]. Regarding an effective cellular immune response the presence or absence of effector and regulatory T-cells is essential. According to the cytokine profile CD4+ T-cells can be divided in Th1 (T-helper 1)-cells, characterised by the secretion of IFN-γ, in Th2 (T-helper 2)-cells, characterised by the secretion of IL-4/IL-5 and in so called Tr1 cells, characterized by the secretion of IL-10. Furthermore TGF-β secretion is the main characteristic of Th3 cells [7,8]. Basically, Th1-cells activate cytotoxic CD8+ T-cells thus causing an antitumoral effect whereas Th2/Th3/Tr1-cells boost the tumor progression by secretion of the above mentioned cytokines. The change of a Th-1 cell mediated cytokine profile to a profile typical for Th2/Th3/Tr1-cells is called "Th-1/Th-2 shift" and is considered a major cause of an ineffective cellular immune response during carcinogenesis [9]. Furthermore, regulatory CD4+CD25+ T-cells (Tregs) which express the transcriptional repressor gene Foxp3 and emerge as mature T cells from the thymus, are increasingly under consideration in regard of the ineffectiveness of the cellular immune response to malignant cells [10-12]. Tregs play a critical role in maintaining tolerance to self antigen and in preventing autoimmunity [13]. Inducible Tregs are generated in the periphery in response to pathogens and self antigens [7,14]. However, the induction or activation of Tregs by pathogens may be one strategy to subvert protective immunity whereas depletion of CD4+CD25+ Tregs enhances survival during certain infections [15]. Under certain conditions, typically following exposure to IL-10 or TGF-β, dendritic cells within the tumor microenvironment and cancer cells can initiate the development of Tregs limiting effector responses by inhibition of cytotoxic T-cells thus impairing anti tumor immunity [16,17]. In conclusion, TGF-β and IL-10 expression is intimately implicated in tumor development and contributes to many features of tumor cell biology.

In this study we used an intraportal liver metastases animal model with CT26.WT colon cancer cells for the time-dependent analysis of immunological tumor escape mechanisms during carcinogenesis considering the above mentioned cytokine and cellular interactions.

## Methods

### Animals

Female Balb/c mice, 6-8 weeks of age, were purchased from Harlan-Winkelmann (Borchem, Germany). Animals were maintained under conventional housing conditions and under specific pathogen-free conditions. Regular screenings for the presence of murine pathogens (such as Mycoplasma pulmonis, Streptococcus pneumoniae, and Helicobacter spp.) were performed. All mice were fed a commercial diet, given water ad libitum, and subjected to an equal 12-hour light/dark cycle in accordance with institutional guidelines. Housing and all procedures involving animals were performed according to protocols approved by the University's animal care committee and in compliance with the guidelines on animal welfare of the National Committee for Animal Experiments.

### Cell line

CT26 is an N-nitroso-N-methylurethane-(NNMU) induced, undifferentiated murine colon carcinoma cell line, which was cloned to generate the cell line designated CT26.WT (LGC promochem, Germany). This cell line is tumorigenic and induces colon carcinoma in rodents [18]. CT26.WT cells were propagated and subcultured according to the distributor's protocol. The cultures were routinely tested for mycoplasma contamination to ensure that only negative cells were used.

### Surgical procedure

The surgical procedure of intraportal tumor cell injection has already been described extensively [19]. 1 × 10^5 ^tumor cells in 100 μl PBS were injected into the portal vein using a 32G needle. In all control group animals a laparotomy and an intraportal injection of 100 μl PBS solution without tumor cells was performed (n = 3). Mice were sacrificed at 5, 10, 15, and 20 days after injection (n = 7 mice per group) or earlier if tumor related cachexy or hepatic failure with tumor related ascites occurred. Control group animals were sacrificed on day 5 following PBS injection. Liver and lung tissues of all animals were collected and subjected to histological examination. Liver metastases were further examined by immunohistochemistry, Real Time PCR, and TUNEL assay. All surgical procedures administered to the animals were in accordance with institutional guidelines. Tumor volumes were calculated by measurements of the short and long axis of the mass of the prominent metastasis, where V = π/6 × a^2 ^× b (a: short axis of the tumor, b: long axis of the tumor).

### Immunohistochemistry/Immunofluorescence

About half of the complete liver tissue was fixed in 4% buffered formalin for histological examination and PCR-analysis. Paraffin sections were evaluated using hematoxylin and eosin. The rest of the liver tissue was either snap-frozen and stored at -80°C for RNA extraction and Real Time PCR analysis or fixed in acetone for Cryostat sections.

Monoclonal antibodies (mAb) were purchased as follows: anti-CD4-mAb (MCA 1767), anti-CD8-mAb (MCA 609G), anti-CD25-mAb (14-0251), anti-Foxp3-mAb (14-5773) from eBioscience (San Diego, CA, USA), anti-FAS-L-mAb (ab21233) and anti-FAS-mAb (321-335 Cat. No. PC69) from Calbiochem (Darmstadt, Germany). Cy3 conjugated anti-CD4-mAb and anti-CD8-mAb were purchased from Linaris (Wertheim, Germany). Isotype-matched mAbs or purified IgG1 (MCA1211, Serotec, Oxford, England) and controls for residual endogenous peroxidase activity were included in each experiment.

The analysis of single staining was performed for CD4, Foxp3, CD8, CD25, FAS, and FAS-L. Immunohistochemical double staining was carried out for CD4 and CD8 following standard staining procedures. For immunohistochemical double staining, antibodies bound during the first staining step were eluted using LIN-Block (RAG0149LK) (Linaris, Wertheim, Germany) according to the manufacturer's instructions, and the slides were subsequently incubated with the second primary mAb diluted in TBS plus 0.5% BSA for 20 min at 37°C followed by AP-conjugated secondary antibody and development with Vector Blue (SK-5300) (Linaris) for 30 min and counterstaining with hemalaun for 1 minute.

The sequential immunofluorescence double staining (coexpression) was detected with Foxp3+ and CD4+ and FAS+ with CD8+ cells. The slides were incubated with the primary antibody or control antibody diluted in TBS plus 0.5% bovine serum albumin (BSA) overnight at 4°C in a humidified chamber and with secondary FITC-conjugated (fluorescein isothiocyanate) antibody for 30 minutes at room temperature in a humidified chamber. The slides were blocked with 10% normal rat serum diluted in TBS and incubated with the Cy3 conjugated primary antibody diluted in TBS plus 0.5% BSA overnight at 4°C in a humidified chamber. Slides were counterstained with DAPI (4',6-diamidino-2-phenylindole) if necessary (Sigma-Aldrich, Steinheim, Germany).

The quantification of each immunohistochemical staining was done by cell counting in six individual representative high power fields (× 400). Likewise, the evaluation of immunofluorescent double staining was performed by counting CD4+ or CD8+ cells in six high power fields (×400) together with Foxp3+ or FAS+ cells in cryostat sections. The proportion of Foxp3 positivity in counted CD4+ and the proportion of FAS positivity in counted CD8+ cells were expressed in percentages. The result of the staining was expressed in percentages (%) positivity. Results were examined by two blinded and unbiased colleagues experienced in gastrointestinal pathology and histology.

### RNA extraction

RNA was extracted using an RNA extraction kit (Qiagen, Hilden, Germany) from at least 10 mg of homogenized tumor tissue. After homogenization DEPC-75% ethanol was added to the lysate to provide ideal binding conditions. The lysate was then loaded onto the RNeasy silica membrane ("RNeasy Mini spin column"). After binding of RNA all contaminants, including genomic DNA, were efficiently washed out. Pure, concentrated RNA was eluted in water and stored at -70°C until further analyses. The amount of total RNA was determined by measuring absorbance at 260 nm. The purity of the total RNA was established by confirming that the 260 nm: 280 nm ratio was within a 1.8-2.0 range, indicating that the RNA preparations were free of protein contaminants.

### RT-PCR for cytokine genes in metastatic tumor specimens

mRNA expression of representative surface molecules and cytokines (CD4, CD8, CD25, Foxp3, IL-10, TGF-β, TNF-α, IFN-γ, FAS, and FAS-L) was analyzed in metastatic tumor specimens by Real Time PCR. Experiments were done 4-6 times per animal. RNA was extracted as described above. cDNA was prepared using 2 μg of heat-denatured RNA. Primer sets from Quiagen (Hilden, Germany) were used for analysis. Optimum primer concentration was determined by titration. Real Time quantitative PCR was performed in a two-step RT-PCR using SYBR-Green PCR Master Mix (PE Biosystems, Foster City, CA) with 100 ng cDNA and 300 nM of primers in a total reaction volume of 50 μl. PCR thermal cycling conditions were as follows: 95°C for 10 min, followed by 40 cycles of 95°C for 15 sec and 60°C for 60 sec. Gene specific products were continuously measured by an ABI PRISM 7700 sequence detector (Applied Biosystems, Foster City, CA) and relative quantification was performed following the manufacturer's instructions.

### *In situ *detection of apoptosis

To identify cells with fragmented DNA we used a nonisotopic DNAend-labeling in situ technique, employing digoxigenin-dNTP and terminal transferase (ApopTag^® ^Fluorescein in Situ Apoptosis Kit, Chemicon, Planegg-Muenchen, Germany). Briefly, sections were post-fixed and equilibrated in terminal transferase buffer before the addition of reaction buffer containing digoxigenin-dNTP oligonucleotide. The digoxigenin-dNTP-containing oligonucleotide extensions were detected by anti-digoxigenin-fluorescein conjugate diluted in a blocking agent, followed by blocking with 10% normal rat serum diluted in TBS (DAKO) and incubated with the Cy3 conjugated primary antibody anti-CD8-mAb diluted in TBS plus 0.5% BSA overnight at 4°C in a humidified chamber. The sections were washed and covered with Polyvinyl-alcohol mounting medium (DABCO) (Sigma-Aldrich) and analyzed using a Zeiss camera (Jena, Germany). Photographed Images using the Metamorph software (Visitron Systems, Puchheim, Germany) package were imported into Microsoft Office Picture Manager. For negative controls, sections were incubated with TUNEL reaction mixture without TdT. For positive controls, sections of female mammary gland were used.

The apoptotic index (AI) was defined as the ratio of TUNEL-positive infiltrated cells to all counted infiltrated cells × 100. For each group, the number of stained cells was counted in at least ten 400 × high-power fields. Cells were defined as apoptotic if the whole nuclear area of the cell labelled was positive.

### Statistical analysis

Results were expressed as mean ± SD. Each of the above mentioned studies was performed in seven mice per group. Comparisons were performed by analysis of variance or paired and unpaired *t *test when appropriate. Bonferroni's correction for multiple comparisons was used to determine the level of significance. *p *< 0.05 was considered significant.

## Results

### Induction of liver metastases by intraportal injection of CT26.WT colon carcinoma cells

The injection of 1 × 10^5 ^CT26.WT cells in 100 μl PBS-solution resulted in development of hepatic metastases in all animals. There was a continuous increase in tumor volume overtime (day 5: 8.4 ± 2 mm^3^, day 10: 150.7 ± 32 mm^3^, day 15: 435.4 ± 37 mm^3^, day 20 680.3 ± 41 mm^3^). Therefore, the injection of 1 × 10^5 ^CT26.WT cells was used for this study. None of the animals showed pulmonary metastases.

### Analysis of T cell infiltration at early and late tumor stages

**Figure 1:**
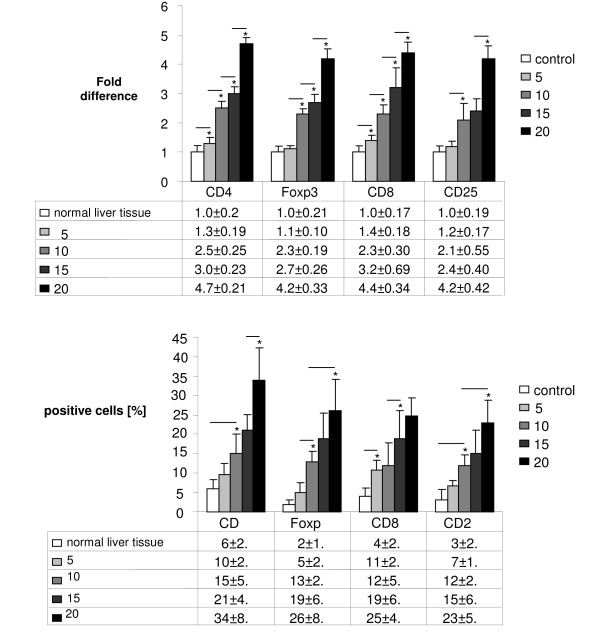
**(a) Real Time PCR: Significantly increasing gene expression of CD4, Foxp3, CD8, and CD25 in metastatic liver tissue on day 5, 10, 15 and 20 after intraportal injection of 1 × 10^5 ^CT26.WT cells (each group n = 7)**. Analysis was measured to control tissue (liver). **(b) **Immunohistochemistry: Increasing number (in %) of CD4, Foxp3, CD8, and CD25 at metastatic sites in the liver over time (day 5, 10, 15 and 20) after intraportal injection of 1 × 10^5 ^CT26.WT cells (each group n = 7). Lines with asterisks show significant increase during metastatic growth.

**Figure 2:**
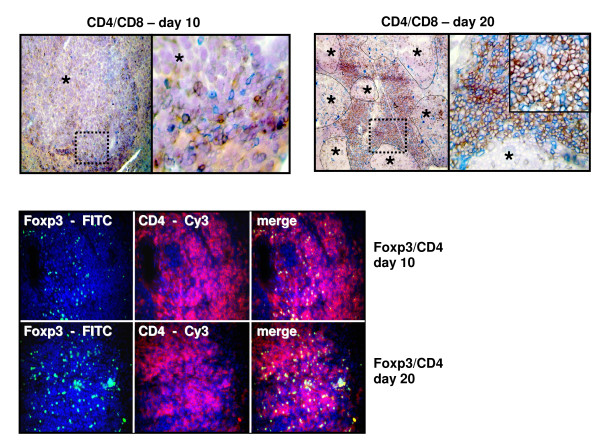
**(a) Immunohistochemistry and (b) immunofluorescence double staining: Representative image of CD4 (red)/CD8 (blue) (top) expression and Foxp3 (FITC)/CD4 (Cy3) (bottom) expression in liver metastases on day 10 (n = 7) compared to increased CD4 (red)/CD8 (blue) expression and Foxp3 (FITC)/CD4 (Cy3) expression on day 20**. Immunohistochemistry: Nova red brick red color, Vector Blue blue color. Haemalaun blue color-nuclear counterstaining. Asterisk with indication lines show metastases next to normal tissue (×100 and ×400). Immunofluorescence: FITC green fluorescein isothiocyanate, Cy3 red and DAPI 4',6-diamidino-2-phenylindole blue - nuclear counterstaining (×250).

Real Time PCR analysis following intraportal injection of 1 × 10^5 ^CT26.WT cells (n = 7 at each time point) showed significantly higher gene expression level of specific T cell markers during metastatic growth. A significant increase of the expression of CD4, Foxp3 and CD8 could be shown at all points of time compared to the previous time segment. Additionally, the expression of CD4 and CD8 at day 5 was already significantly increased compared to control tissue. The expression of CD25 showed a significant increase from day 5 to day 10 and from day 15 to day 20. Relative quantification value, fold difference, is expressed as 2^-ΔΔCt ^(Figure 1a). These findings were immunohistologically confirmed showing an expression pattern according to the increased gene expression of CD4, Foxp3, CD8, and CD25 during formation of liver metastases. Immunohistologically increasing numbers of Foxp3+ T cells as well as CD4, CD25, and CD8 were detected during carcinogenesis (Figure 1b). Especially the number of CD4+ T cells increased over proportionally on day 20 post injectionem (Figure 2a). Using immunofluorescence Foxp3+ expressing cells were identified as CD4+ phenotype which were increased at day 20 compared to day 10 (Figure 2b).

### Cytokine expression

**Figure 3:**
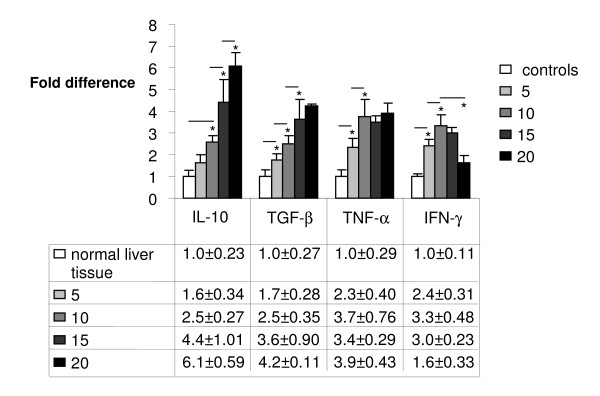
**Real Time PCR: Significantly increasing gene expression of IL-10, TGF-β, TNF-α but a decrease of IFN-γ at metastatic sites in the liver on day 10, 15 and 20 after intraportal injection of 1 × 10^5 ^CT26.WT cells**. Analysis was measured to control tissue (liver). Lines with asterisks show significant differences during metastatic growth.

Following injection of 1 × 10^5 ^CT26.WT cells Real Time PCR analysis showed significantly increasing gene expression of IL-10 from day 10 to day 20. The expression of TGF-β was significantly increased on day 10 and 15 compared to the previous point of time, whereas TNF-α showed a significant increase in expression only until day 10. From this time on no significant changes occurred with TNF-α. In contrast, IFN-γ gene expression increased significantly until day 10. From day 10 on, however, a significant decrease of expression could be observed until day 20 (Figure 3).

### Analysis of FAS/FAS-L expression on tumor cells during metastatic growth

**Figure 4:**
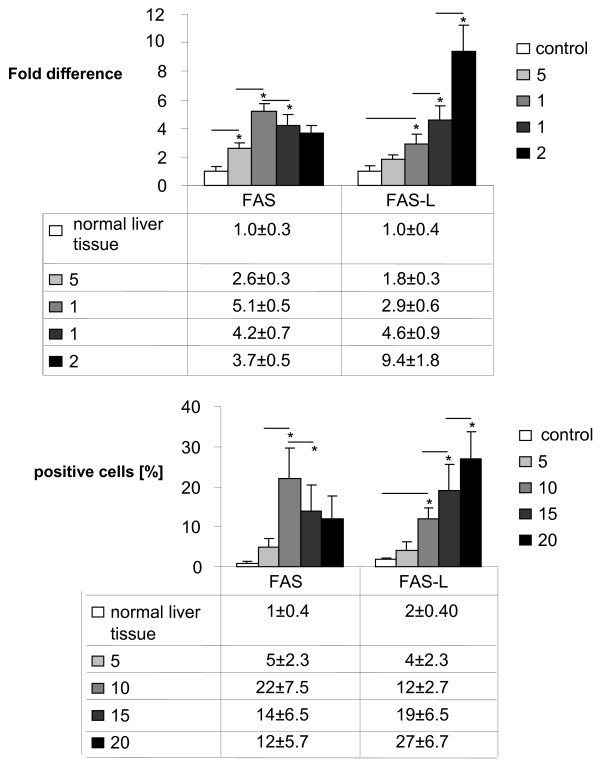
**(a) Real Time PCR: Downregulation of FAS gene expression from day 10 on following an initial increase but steadily increasing of FAS-L gene expression in metastatic liver over time (day 5, 10, 15 and 20) following intraportal injection of 1 × 10^5 ^CT26.WT cells**. Analysis was measured to control tissue (liver). **(b) **Immunohistochemistry: Increasing number (in %) of FAS-L expressing tumor cells but a decreased FAS expression from day 10 on following intraportal injection of 1 × 10^5 ^CT26.WT cells.

In Real Time PCR analysis, down regulation of FAS gene expression in metastatic liver tissue could be detected from day 10 on (p < 0.05 day 10 to day 15) following an initial increase in FAS gene expression at day 5 and 10. FAS-L expression was increased during tumor development (p < 0.05, day 10 compared to day 15 and day 20 respectively, Asterisk) (Figure 4a). Immunohistologically FAS-L expression was morphologically attributed to the tumor cells in liver metastases at late tumor stages (p < 0.05, day 10 compared to day 15, day 20 and normal tissue, respectively, Asterisk; Figure 4b, 5a). Additionally the immunohistological detection of FAS receptor in liver metastases showed a significant decrease from day 10 to day 15 following an initial increase in expression (Fig 4b, 5b). The expression of FAS and FAS-L by tumor cells was confirmed by positive control staining of the tumor cell line CT.26 WT. FAS expression was observed predominantly on CD8+ T cells infiltrating the perimetastatic margin: CD8+/FAS day 10: 12 ± 4.9%, day 15: 15 ± 5.4%, day 20: 21 ± 4.7% (day 20 shown in Figure 6a). TUNEL assay confirmed apoptotic events in these perimetastatic areas and showed an increased rate of apoptotic CD8+ cells during tumor growth: CD8+/TUNEL day 10: 5.6 ± 2.9%, day 15: 13 ± 4.7%, day 20: 20 ± 3.4% (day 20 shown in Figure 6b).

**Figure 5:**
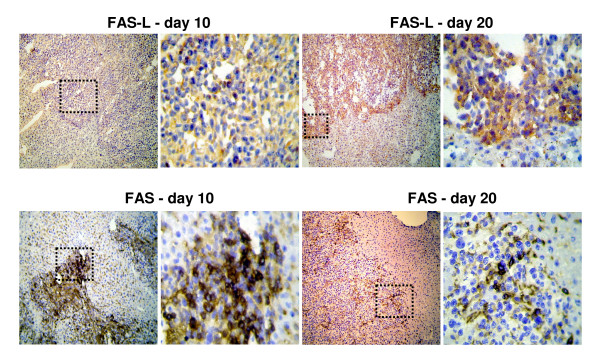
**(a) Immunohistochemistry: Representative images of FAS-L expression in liver metastases on day 10 (left) compared to increased FAS-L expression on day 20 (right)**. **(b) **Expression of FAS in liver metastasis on day 10 compared to day 20 demonstrating a decreased expression in the course of metastatic growth. DAB (3,3'-diaminobenzidine) brown color, Haemalaun blue color - nuclear counterstaining. Magnification ×100 and ×400. Case demonstrates area of magnification.

**Figure 6:**
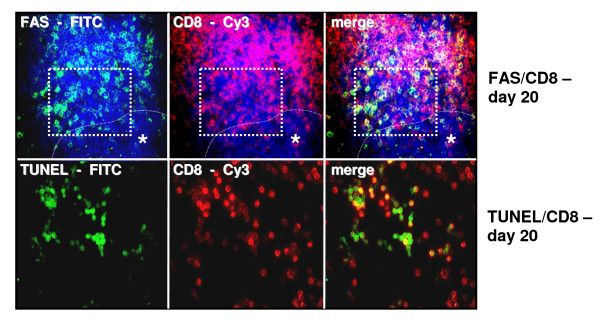
**Immunofluorescence: Representative images of increased CD8+/FAS (a) and CD8+/TUNEL (b) expression on tumor infiltrating lymphocytes at the margin of liver metastases on day 20**. FITC green Fluoresceinisothiocyanat, Cy3 red and DAPI 4',6-Diamidino-2-phenylindoldihydrochlorid blue - nuclear countersaining. Case demonstrates area of the TUNEL staining in serial cryostat sections. To adjust a better contrast TUNEL stained sections were not counterstained with DAPI. Asterisk with indication line shows metastatic tumor cells. Magnifications ×250 (top) and ×400 (bottom).

## Discussion

Based on the hypothesis of the immunogenic effect of malignant transformed cells, firstly announced in 1957 by Sir Frank MacFarlane Burnet, lymphocytes have a central role in cancer immunosurveillance [20]. Nowadays this hypothesis is proven although the interaction between malignant tumors and the immune system is much more complex than Mr Burnet thought more than fifty years ago. Increasing knowledge of the interplay between malignant cells and the innate and adaptive immune system is the basis for the development of modern immunotherapeutic strategies. These strategies, however, need to be tested preclinically in reliable animal models.

In this study we describe a colorectal liver metastases animal model showing tumor growth in an immunocompetent mouse. In such a model tumor induced immune responses similar to the situation in human beings can be analysed at different points of time during carcinogenesis. In this regard such a model can be an important tool for further immunotherapeutic intervention studies.

CT26.WT, which has been used in this study, is a common murine colon cancer cell line, which has been used in several studies to investigate the mechanisms of tumor stromal interactions and anti-tumor immune responses [21,22]. Numerous animal models have been developed to gain detailed information on initiation, promotion, and progression of colorectal liver metastases. The morphology, biochemical alterations, and biological behaviour of the malignant disease should be, compared to the situation in humans, reproduced properly by an animal model. Thus, the main purpose of such a model should be to approximate as much characteristics as possible of the emergence of liver metastases. We are fully aware of the fact that our model does not represent the initial steps of dissociation, invasion and intravasation of the metastatic process [23]. Nonetheless, taking into account the prompt and reproducible initiation of a constant number of liver metastases following intraportal injection of CT26.WT colon cancer cells, the herein described model fulfils the demands of a sufficient and biological reliable animal model requested for the study of new therapeutical approaches.

The cellular invasion with predominantly lymphocytes infiltrating the tumor is one of the main features of the host immune response. The presence of tumor specific T cells has been correlated with improved clinical outcome in different human cancers [24,25], but does not necessarily result in anti-tumor immunity since T cells can also promote the progression of tumors through different growth factors [26]. In this regard high densities of CD3+ tumor infiltrating T cells in node negative colorectal cancer were shown to make the occurrence of metachrone metastases unlikely [27]. Furthermore, high densities of CD8+/granzymeB+ T cells and CD45+ memory T cells at the tumor margin and in the center of colorectal cancers were found to be a better predictor of patient survival than the histopathological methods usually used, leading to the hypothesis that not only the type but also density and location of tumor infiltrating T cells may be a more critical determinant for the prognosis [28,29]. In this study we observed a steadily increasing number of CD4+ and CD8+ T cells beleaguering the liver metastases. In contrast to human colon cancer in which the rate of tumor infiltrating lymphocytes is less than 10% of surrounding stromal cells [30], we found a much higher number of primarily CD4+ T cells. This has to be attributed to an obviously different cellular immune response in this rodent model as the quantity of tumor infiltrating lymphocytes seen in this study obviously does not reflect the human situation. The fact that most CD4+ T cells were also positive for Foxp3 shows that at different points of time during carcinogenesis in this model, an increasing number of phenotypical regulatory T cells (Tregs) were detectable at the tumor site demonstrating a potential protumoral effect. This is in accordance with data showing that natural CD4+CD25+ Tregs may play a critical role in the progression of a number of cancers by suppressing anti tumor immune response effects [31-33]. In this regard we previously demonstrated that an increasing number of Tregs specific genes at the tumor site correlate with the UICC stage of human colon cancer supporting the protumoral effect of Tregs [10]. By contrast, however, it was shown recently that a high density of Foxp3+ Tregs in tumor tissue of patients with colon cancer was associated with an improved survival [34]. Thus, the final assessment of a potential anti- or protumoral effect of Tregs in colon cancer can still not be served. Recently a new CD8+CD25+Foxp3+ (T8reg) T cell subset was described in patients with colon cancer [35]. Although in small absolute number, T8regs were more abundant in the blood and tumor tissue of tumor patients compared to healthy volunteers. The study provides indirect evidence for an inhibitory effect of T8regs on the antitumor immune response which seems to be similar to the effects mediated by CD4+CD25+ Tregs. In our study we did not analyze the presence of T8regs which however should be of interest in further studies.

CD4+ T cell response for anti-tumor immunity can be divided into different types depending upon their cytokine profile [36]. Th1 cells are characterized especially by the production of IFN-γ, whereas Th2, Th3 and Tr1 cells secret cytokines IL-4, IL-5, IL-10 and TGF-β. The balance between Th1 and Th2/Th3/Tr1 cytokines has definite influence on the outcome of various immune responses, as Th1 preferentially induces cellular immunity whereas the others tend to elicit humoral immunity. Basically a Th1 cytokine profile mediates an anti-tumor immunity whereas a Th2/Th3/Tr1 cytokine profile supports a pro-tumor activity. The change from a Th1 profile to a Th2/Th3/Tr1 profile, the so called Th1-/Th2-shift, is an important feature of an insufficient cellular immunity during carcinogenesis [37,38]. In colon cancer the expression of Th1-associated genes like IFN-γ, which exerts antiproliferative, antiangiogenic, and proapoptotic effects on a variety of tumor cells [39,40], was significantly associated with a more favourable clinical course of the disease [41].

In our tumor animal model described in this study cytokines IL-10 and TGF-β were expressed increasingly during carcinogenesis at different points of time in metastatic liver tissue following intraportal CT26.WT colon carcinoma cell injection whereas the expression of IFN-γ decreased beginning from day 10 after tumor cell injection. These results are in accordance to previous studies [4] and reflect the above mentioned Th1-/Th2 shift which could be observed in our model in the later stages of carcinogenesis (day 10 to 20). TNF-α, which is an important proinflammatory cytokine produced mainly by macrophages and dendritic cells, is known to support the development of IL-10 producing Tr1 cells [42]. Thereby the protumoral cytokine profile is augmented additionally by increasing levels of TNF-α, which also has been demonstrated in a murine melanoma model [43]. Thus, in this model the pathophysiological change of cytokine profiles seen in human reality seems to be imitated properly.

It has been shown that the interaction between FAS and its ligand (FAS-L) induced FAS-positive cell apoptosis in colorectal tumor cell lines [44]. Different strategies of tumors to escape cancer immunosurveillance involving these molecules have been studied and led to the so called "counterattack" hypothesis [45,46]. We observed that FAS gene expression was down regulated in liver metastases beginning from day 10 after tumor cell injection following an initially increased expression. FAS-L expression was increasingly detected at all points of time during carcinogenesis. In addition, FAS-L expression was clearly attributed to the tumor cell surface in liver metastases shown by immunohistochemistry, while FAS expression was observed predominantly on CD8+ T cells infiltrating the perimetastatic site. Proper to this end the number of apoptotic CD8+ T cells was increased time-dependent during growth of metastases. This might contribute to the impairment of the cellular anti-tumor immune response although elevated gene expression and immunohistological confirmation of an increasing number of CD8+ T cells was demonstrated during carcinogenesis in our model. This result supports the "counterattack" hypothesis morphologically and implies that tumor cells possibly circumvent immunological surveillance by up-regulation of FAS-L expression and down-regulation of FAS, at least at later stages of carcinogenesis. The "counterattack" hypothesis, however, is hard to prove in vivo, since we cannot demonstrate the functional significance of these findings by mere morphological observation.

## Conclusion

In conclusion, this study describes immunological escape mechanisms during metastatic tumor growth in a colorectal liver metastases mouse model at different points in time. We have shown that tumor growth induced an extensive cellular immune response of predominantly regulatory T cells with a presumably protumoral activity. Furthermore we could demonstrate a shift from Th1- to Th2-associated cytokine profiles during carcinogenesis simulating the situation in human cancer. Additionally a further feature of tumor immune escape mechanisms, the FAS/FAS-L "counterattack", could be shown in the described murine model.

Taken together this model simulates several features of immunological escape mechanisms during carcinogenesis and might serve as a model for immunotherapeutic intervention studies.

## Competing interests

The authors declare that they have no competing interests.

## Authors' contributions

MG was responsible for immunohistochemical analysis and drafting of the manuscript. MG and MB participated in the surgical animal procedures and the surveillance of operated mice. JS, JW and EN assisted in surgical animal procedures, performed the RT-PCR experiments and participated in data analysis. DM was involved in establishing microsurgical techniques. CTG and AMWG participated in the design of the study and its coordination. AT was responsible for the interpretation of all data and drafting of the manuscript.  All authors read and approved the final manuscript.

## Pre-publication history

The pre-publication history for this paper can be accessed here:

http://www.biomedcentral.com/1471-2407/10/82/prepub

## References

[B1] Compton CC Fielding LP Burgart LJ Conley B Cooper HS Hamilton SR Hammond ME Henson DE Hutter RV Nagle RB Nielsen ML Sargent DJ Taylor CR Welton M Willett C Prognostic Factors in Colorectal Cancer. College of American Pathologists Consensus Statement 1999 Arch Pathol Lab Med 2000 7 979 994 10.5858/2000-124-0979-PFICC 10888773

[B2] Scheele J Stangl R Altendorf-Hofmann A Hepatic Metastases From Colorectal Carcinoma: Impact of Surgical Resection on the Natural History Br J Surg 1990 11 1241 1246 10.1002/bjs.1800771115 2253003

[B3] Lorenz M Staib-Sebler E Hochmuth K Heinrich S Gog C Vetter G Encke A Muller HH Surgical Resection of Liver Metastases of Colorectal Carcinoma: Short and Long-Term Results Semin Oncol 2000 5 Suppl 10 112 119 11049042

[B4] Jarnicki AG Lysaght J Todryk S Mills KH Suppression of Antitumor Immunity by IL-10 and TGF-Beta-Producing T Cells Infiltrating the Growing Tumor: Influence of Tumor Environment on the Induction of CD4+ and CD8+ Regulatory T Cells J Immunol 2006 2 896 904 10.4049/jimmunol.177.2.896 16818744

[B5] Elkord E Hawkins RE Stern PL Immunotherapy for Gastrointestinal Cancer: Current Status and Strategies for Improving Efficacy Expert Opin Biol Ther 2008 4 385 395 10.1517/14712598.8.4.385 18352844

[B6] Khong HT Restifo NP Natural Selection of Tumor Variants in the Generation of "Tumor Escape" Phenotypes Nat Immunol 2002 11 999 1005 10.1038/ni1102-999 PMC1508168 12407407

[B7] Kosmaczewska A Ciszak L Potoczek S Frydecka I The Significance of Treg Cells in Defective Tumor Immunity Arch Immunol Ther Exp (Warsz) 2008 3 181 191 10.1007/s00005-008-0018-1 18512029

[B8] Oldenhove G de Heusch M Urbain-Vansanten G Urbain J Maliszewski C Leo O Moser M CD4+ CD25+ Regulatory T Cells Control T Helper Cell Type 1 Responses to Foreign Antigens Induced by Mature Dendritic Cells in Vivo J Exp Med 2003 2 259 266 10.1084/jem.20030654 PMC2194073 12874259

[B9] Cui G Florholmen J Polarization of Cytokine Profile From Th1 into Th2 Along Colorectal Adenoma-Carcinoma Sequence: Implications for the Biotherapeutic Target? Inflamm Allergy Drug Targets 2008 2 94 97 10.2174/187152808785107589 18691138

[B10] Bueter M Gasser M Schramm N Lebedeva T Tocco G Gerstlauer C Grimm M Nichiporuk E Thalheimer A Thiede A Meyer D Benichou G Waaga-Gasser AM T-Cell Response to P53 Tumor-Associated Antigen in Patients With Colorectal Carcinoma Int J Oncol 2006 2 431 438 16391798

[B11] Golgher D Jones E Powrie F Elliott T Gallimore A Depletion of CD25+ Regulatory Cells Uncovers Immune Responses to Shared Murine Tumor Rejection Antigens Eur J Immunol 2002 11 3267 3275 10.1002/1521-4141(200211)32:11<3267::AID-IMMU3267>3.0.CO;2-1 12555672

[B12] Woo EY Chu CS Goletz TJ Schlienger K Yeh H Coukos G Rubin SC Kaiser LR June CH Regulatory CD4(+)CD25(+) T Cells in Tumors From Patients With Early-Stage Non-Small Cell Lung Cancer and Late-Stage Ovarian Cancer Cancer Res 2001 12 4766 4772 11406550

[B13] Sakaguchi S Regulatory T Cells: Key Controllers of Immunologic Self-Tolerance Cell 2000 5 455 458 10.1016/S0092-8674(00)80856-9 10850488

[B14] Mills KH Regulatory T Cells: Friend or Foe in Immunity to Infection? Nat Rev Immunol 2004 11 841 855 10.1038/nri1485 15516964

[B15] Hisaeda H Maekawa Y Iwakawa D Okada H Himeno K Kishihara K Tsukumo S Yasutomo K Escape of Malaria Parasites From Host Immunity Requires CD4+ CD25+ Regulatory T Cells Nat Med 2004 1 29 30 10.1038/nm975 14702631

[B16] Yu P Fu YX Tumor-Infiltrating T Lymphocytes: Friends or Foes? Lab Invest 2006 3 231 245 10.1038/labinvest.3700389 16446705

[B17] Jakowlew SB Transforming Growth Factor-Beta in Cancer and Metastasis Cancer Metastasis Rev 2006 3 435 457 10.1007/s10555-006-9006-2 16951986

[B18] Kollmar O Schilling MK Menger MD Experimental Liver Metastasis: Standards for Local Cell Implantation to Study Isolated Tumor Growth in Mice Clin Exp Metastasis 2004 5 453 460 10.1007/s10585-004-2696-3 15672870

[B19] Thalheimer A Otto C Bueter M Illert B Gattenlohner S Gasser M Meyer D Fein M Germer CT Waaga-Gasser AM The Intraportal Injection Model: a Practical Animal Model for Hepatic Metastases and Tumor Cell Dissemination in Human Colon Cancer BMC Cancer 2009 29 10.1186/1471-2407-9-29 19166621 PMC2648996

[B20] Burnet M Cancer: a Biological Approach. III. Viruses Associated With Neoplastic Conditions. IV. Practical Applications Br Med J 1957 5023 841 847 10.1136/bmj.1.5023.841 PMC1973618 13413231

[B21] Chan WS Page CM Maclellan JR Turner GA The Growth and Metastasis of Four Commonly Used Tumour Lines Implanted into Eight Different Sites: Evidence for Site and Tumour Effects Clin Exp Metastasis 1988 3 233 244 10.1007/BF01782483 3349666

[B22] Isbert C Boerner A Ritz JP Schuppan D Buhr HJ Germer CT In Situ Ablation of Experimental Liver Metastases Delays and Reduces Residual Intrahepatic Tumour Growth and Peritoneal Tumour Spread Compared With Hepatic Resection Br J Surg 2002 10 1252 1259 10.1046/j.1365-2168.2002.02205.x 12296892

[B23] Chambers AF Groom AC MacDonald IC Dissemination and Growth of Cancer Cells in Metastatic Sites Nat Rev Cancer 2002 8 563 572 10.1038/nrc865 12154349

[B24] Zhang L Conejo-Garcia JR Katsaros D Gimotty PA Massobrio M Regnani G Makrigiannakis A Gray H Schlienger K Liebman MN Rubin SC Coukos G Intratumoral T Cells, Recurrence, and Survival in Epithelial Ovarian Cancer N Engl J Med 2003 3 203 213 10.1056/NEJMoa020177 12529460

[B25] Molldrem JJ Lee PP Wang C Felio K Kantarjian HM Champlin RE Davis MM Evidence That Specific T Lymphocytes May Participate in the Elimination of Chronic Myelogenous Leukemia Nat Med 2000 9 1018 1023 10.1038/79526 10973322

[B26] Inagaki A Ishida T Ishii T Komatsu H Iida S Ding J Yonekura K Takeuchi S Takatsuka Y Utsunomiya A Ueda R Clinical Significance of Serum Th1-, Th2- and Regulatory T Cells-Associated Cytokines in Adult T-Cell Leukemia/Lymphoma: High Interleukin-5 and -10 Levels Are Significant Unfavorable Prognostic Factors Int J Cancer 2006 12 3054 3061 10.1002/ijc.21688 16425276

[B27] Laghi L Bianchi P Miranda E Balladore E Pacetti V Grizzi F Allavena P Torri V Repici A Santoro A Mantovani A Roncalli M Malesci A CD3+ Cells at the Invasive Margin of Deeply Invading (PT3-T4) Colorectal Cancer and Risk of Post-Surgical Metastasis: a Longitudinal Study Lancet Oncol 2009 9 877 884 10.1016/S1470-2045(09)70186-X 19656725

[B28] Curiel TJ Coukos G Zou L Alvarez X Cheng P Mottram P Evdemon-Hogan M Conejo-Garcia JR Zhang L Burow M Zhu Y Wei S Kryczek I Daniel B Gordon A Myers L Lackner A Disis ML Knutson KL Chen L Zou W Specific Recruitment of Regulatory T Cells in Ovarian Carcinoma Fosters Immune Privilege and Predicts Reduced Survival Nat Med 2004 9 942 949 10.1038/nm1093 15322536

[B29] Galon J Costes A Sanchez-Cabo F Kirilovsky A Mlecnik B Lagorce-Pages C Tosolini M Camus M Berger A Wind P Zinzindohoue F Bruneval P Cugnenc PH Trajanoski Z Fridman WH Pages F Type, Density, and Location of Immune Cells Within Human Colorectal Tumors Predict Clinical Outcome Science 2006 5795 1960 1964 10.1126/science.1129139 17008531

[B30] Michael-Robinson JM Pandeya N Walsh MD Biemer-Huttmann AE Eri RD Buttenshaw RL Lincoln D Clouston AD Jass JR Radford-Smith GL Characterization of Tumour-Infiltrating Lymphocytes and Apoptosis in Colitis-Associated Neoplasia: Comparison With Sporadic Colorectal Cancer J Pathol 2006 3 381 387 10.1002/path.1895 16315333

[B31] Clarke SL Betts GJ Plant A Wright KL El Shanawany TM Harrop R Torkington J Rees BI Williams GT Gallimore AM Godkin AJ CD4+CD25+FOXP3+ Regulatory T Cells Suppress Anti-Tumor Immune Responses in Patients With Colorectal Cancer PLoS One 2006 e129 10.1371/journal.pone.0000129 17205133 PMC1762416

[B32] Loddenkemper C Schernus M Noutsias M Stein H Thiel E Nagorsen D In Situ Analysis of FOXP3+ Regulatory T Cells in Human Colorectal Cancer J Transl Med 2006 52 10.1186/1479-5876-4-52 17166272 PMC1764431

[B33] Casares N Arribillaga L Sarobe P Dotor J Lopez-Diaz dC Melero I Prieto J Borras-Cuesta F Lasarte JJ CD4+/CD25+ Regulatory Cells Inhibit Activation of Tumor-Primed CD4+ T Cells With IFN-Gamma-Dependent Antiangiogenic Activity, As Well As Long-Lasting Tumor Immunity Elicited by Peptide Vaccination J Immunol 2003 11 5931 5939 10.4049/jimmunol.171.11.5931 14634104

[B34] Salama P Phillips M Grieu F Morris M Zeps N Joseph D Platell C Iacopetta B Tumor-Infiltrating FOXP3+ T Regulatory Cells Show Strong Prognostic Significance in Colorectal Cancer J Clin Oncol 2009 2 186 192 10.1200/JCO.2008.18.7229 19064967

[B35] Chaput N Louafi S Bardier A Charlotte F Vaillant JC Menegaux F Rosenzwajg M Lemoine F Klatzmann D Taieb J Identification of CD8+CD25+Foxp3+ Suppressive T Cells in Colorectal Cancer Tissue Gut 2009 4 520 529 10.1136/gut.2008.158824 19022917

[B36] Mosmann TR Sad S The Expanding Universe of T-Cell Subsets: Th1, Th2 and More Immunol Today 1996 3 138 146 10.1016/0167-5699(96)80606-2 8820272

[B37] Romagnani S The Th1/Th2 Paradigm Immunol Today 1997 6 263 266 10.1016/S0167-5699(97)80019-9 9190109

[B38] Cui G Florholmen J Polarization of Cytokine Profile From Th1 into Th2 Along Colorectal Adenoma-Carcinoma Sequence: Implications for the Biotherapeutic Target? Inflamm Allergy Drug Targets 2008 2 94 97 10.2174/187152808785107589 18691138

[B39] Beatty G Paterson Y IFN-Gamma-Dependent Inhibition of Tumor Angiogenesis by Tumor-Infiltrating CD4+ T Cells Requires Tumor Responsiveness to IFN-Gamma J Immunol 2001 4 2276 2282 10.4049/jimmunol.166.4.2276 11160282

[B40] Ikeda H Old LJ Schreiber RD The Roles of IFN Gamma in Protection Against Tumor Development and Cancer Immunoediting Cytokine Growth Factor Rev 2002 2 95 109 10.1016/S1359-6101(01)00038-7 11900986

[B41] Pages F Berger A Camus M Sanchez-Cabo F Costes A Molidor R Mlecnik B Kirilovsky A Nilsson M Damotte D Meatchi T Bruneval P Cugnenc PH Trajanoski Z Fridman WH Galon J Effector Memory T Cells, Early Metastasis, and Survival in Colorectal Cancer N Engl J Med 2005 25 2654 2666 10.1056/NEJMoa051424 16371631

[B42] Hirata N Yanagawa Y Satoh M Ogura H Ebihara T Noguchi M Matsumoto M Togashi H Seya T Onoe K Iwabuchi K Dendritic Cell-Derived TNF-Alpha Is Responsible for Development of IL-10-Producing CD4+ T Cells Cell Immunol 2010 1 37 41 10.1016/j.cellimm.2009.10.009 19931858

[B43] Vohra N Verhaegen M Martin L Mackay A Pilon-Thomas S TNF-Alpha-Treated DC Exacerbates Disease in a Murine Tumor Metastasis Model Cancer Immunol Immunother 2009 in press 19921187 10.1007/s00262-009-0793-5 PMC2839058

[B44] Radfar S Martin H Tilkin-Mariame AF [Tumor Escape Mechanism Involving Fas and Fas-L Molecules in Human Colorectal Tumors] Gastroenterol Clin Biol 2000 12 1191 1196 11173732

[B45] Ryan AE Shanahan F O'Connell J Houston AM Fas Ligand Promotes Tumor Immune Evasion of Colon Cancer in Vivo Cell Cycle 2006 3 246 249 10.4161/cc.5.3.2413 16418579

[B46] Sheehan KM O'Donovan DG Fitzmaurice G O'Grady A O'Donoghue DP Sheahan K Byrne MF Conroy RM Kay EW Murray FE Prognostic Relevance of Fas (APO-1/CD95) Ligand in Human Colorectal Cancer Eur J Gastroenterol Hepatol 2003 4 375 380 10.1097/00042737-200304000-00007 12655257

